# The utility of computed tomography at a district-level public hospital in Cape Town

**DOI:** 10.4102/safp.v66i1.5891

**Published:** 2024-05-27

**Authors:** Ridwaan Osman, Amy Fouten, Nihaad Jacobs, Fawwaz Cader, Francois Ehlers, Nazrana Zalgaonkir, Elaine Erasmus, Daniël van Hoving

**Affiliations:** 1 Department of Health, Faculty of Emergency Medicine, Khayelitsha District Hospital, Cape Town, South Africa; 2 Division of Emergency Medicine, Stellenbosch University, Cape Town, South Africa

**Keywords:** computed tomography, emergency medicine, family medicine, primary healthcare, radiology, district hospital, yield

## Abstract

**Background:**

Computed tomography (CT) has become an invaluable aid in medical diagnostic workup, and its global usage has been shown to be consistently increasing across all departments. While typically located in regional or central hospitals in South Africa, its recent introduction at the district level has many foreseeable benefits. We evaluated its utility at one of the first district hospitals in the Western Cape to obtain a CT suite.

**Objectives:**

This study aimed to describe the type of CT scans ordered, the clinical indications, the prevalence of significant abnormal findings and the agreement between the clinical opinion and radiological diagnosis.

**Methods:**

A descriptive cross-sectional study was conducted over a 1-year period at Khayelitsha Hospital, an entry-level hospital just outside of Cape Town.

**Results:**

A total of 3242 CT scans were analysed. The mean age of patients was 46 years; 51.4% were males. A mean of 13 scans were performed per working day. The head and neck area were the most scanned region (*n* = 1841, 52.3%). Predominantly requested by the Emergency Centre (*n* = 1382, 42.6%), indications were mainly for general medical conditions workup (*n* = 2151, 66.4%). Most scans showed abnormalities (*n* = 2710, 83.6%), with 2115 (65.2%) considered relevant (‘positive yield’). Clinical and CT diagnoses agreed in 1610 (49.7%) cases.

**Conclusion:**

Computed tomography usage at the district level demonstrated positive yield rates comparable to that of tertiary centres. This implies an appropriate utilisation of the service with a potential decrease in the burden on the referral centre.

**Contribution:**

Computed tomography scanners at district-level facilities are appropriately utilised and can provide greater access to care while potentially decreasing the burden on referral centres.

## Introduction

Computed tomography (CT) was first introduced in the medical field in the 1970s and has become an invaluable aid in the diagnostic workup of multiple medical and surgical conditions.^1^ Computed tomography imaging also plays a role in admission patterns, with younger patients being more likely to be discharged after a CT scan and older patients more likely to be admitted.^2^

The use of CT has consistently increased in all medical departments, including the emergency centre. In the United States (US) and Canada, CT imaging in the emergency centre increased by almost 60% between 2005 and 2013.^2^ A similar trend has been noticed in South Africa where CT use has doubled between 2011 and 2013 in certain areas.^3^

The utility of CT scans is well documented with its clinical impact in the emergency centre proven to alter management in 43% of cases.^4^ However, limited data are available with regard to what an appropriate positive yield rate is. A Malaysian audit of CT scans of the head demonstrated a 70% yield rate for non-traumatic cases.^5^ More locally, a study in a tertiary academic hospital in Johannesburg found a 61% positive yield in non-traumatic presentations. It is unclear whether higher yields represent an under-utilisation of CT scanning or if it just reflects effective judicious use.^6^

In 2015, the number of CT scanners available in South Africa was only 1.7 per million people in the public sector, compared with >40 per million people in the US.^7^ Computed tomography scanners are typically situated within regional or central hospitals in the South African public sector,^8^ necessitating the need for district-level hospitals to refer patients for diagnostic imaging. This incurs costs, both monetary through patient transfers and non-monetary due to delays in patient management. A Canadian study indicated that 84% of all patients transferred from a rural facility were specifically to receive a CT scan at the receiving facility.^9^ Similar data are not available in a South African context, but increased availability of CT scanners has the potential benefit of reducing transfer burden, decreasing delays in patient management as well as mitigating the risk of loss to follow up of outpatients because of transport and socio-economic constraints.

In August 2018, Khayelitsha Hospital became one of the first district-level hospitals in the Western Cape province to have an on-site CT scanner,^10^ which runs emergency and elective scans during working hours. The utility of a CT scanner at a district-level healthcare facility should theoretically be huge with many potential benefits, but there is limited data available to support this. This study describes the type, clinical indications and diagnostic yield of CT scans performed at a district-level hospital in Cape Town over a 1-year period. In addition, the prevalence of significant abnormal findings and the congruency between the initial clinical impression and the radiological diagnosis on CT were determined.

## Methods

### Study design

A descriptive cross-sectional study performed at Khayelitsha Hospital for the 1-year period (01 January 2019 – 31 December 2019).

### Study setting

The entry-level hospital is situated just outside of Cape Town, in the expansive township of Khayelitsha, which has a population of over 390 000.^11^ The last official census was in 2001, and this number is likely to be grossly underestimated. The hospital is a 300-bed facility that serves a health district of a predominantly black African population (99%) with high levels of unemployment (38%) and high burden of disease in terms of HIV, tuberculosis and interpersonal violence.^11,12,13,14^ The following specialist departments are present within the hospital: Emergency Medicine, Internal Medicine, General Surgery, Orthopaedics, Psychiatry, Obstetrics and Gynaecology and Paediatrics. In terms of imaging, the hospital is equipped with a 24-h x-ray service, weekday general and obstetric ultrasounds, orthopaedic screening and a CT scanner. Teleradiology is practised, with a radiology consultant and registrar, based at Tygerberg Hospital, assigned to report for Khayelitsha Hospital CTs and x-rays on request, on a rotational basis.

### Study population

All adult patients managed at Khayelitsha Hospital who received a CT scan were eligible for inclusion. Patients were excluded if a CT scan was ordered, but not performed; if the CT scan report was not available; and if no clinical information was present. Incomplete cases were included in the analysis, however, incomplete data points were reported and only excluded from the relevant analyses.

### Data collection

The digital picture archiving and communication system (PACS) of Khayelitsha Hospital was used to filter out patients who received a CT scan over the study period. Relevant data points were extracted from the PACS.

### Variables measured

Collected variables included patient demographics (age, sex), indication for CT scan, type of CT scan performed, date and time CT scan was performed, the specific CT scan findings and the reported CT scan diagnosis.

The indications for CT scan were categorised into diagnostic (either Medical, Trauma or Malignancy), pre-operative planning, staging (for patients with known malignancies) and interval (for patients requiring a follow-up scan). The Diagnostic: Medical category also included all non-trauma surgical-related cases. The type of CT scan performed was categorised according to body region (Head & Neck; Chest; Abdomen & Pelvis; Upper extremity; Lower extremity). An abnormal CT scan included any detected abnormality regardless of whether it was pertinent to the actual diagnosis. The yield of a CT scan was deemed positive when the CT report indicated information that established a diagnosis. The CT scan diagnoses were categorised as normal (no abnormal findings), non-diagnostic (no findings to explain clinical presentation, although there may be abnormal findings) or abnormal according to the applicable ICD-10 (International Statistical Classification of Diseases and Related Health Problems, 10th Revision) diagnostic categories. Where a differential diagnosis was given, the most likely diagnosis was used. Where there were multiple diagnoses, whether in a single or multiregion examination, the diagnosis explaining the clinical question was coded primarily, and the second diagnosis was added as a secondary diagnosis code.

Agreement between the clinical (pre-CT scan) diagnosis and the final CT scan diagnosis was judged to be ‘congruent’, ‘non-congruent’ or ‘not applicable’. Agreement was congruent if the clinical diagnosis was the same as the CT scan diagnosis and non-congruent if the diagnoses were completely different from each other. The not applicable categorisation was used when the scan itself was suboptimal because of technical errors. These categorisations were based on the premise that all CTs were ordered suspecting an abnormality that would explain the clinical presentation. Thus, a normal or non-diagnostic CT would always be non-congruent.

### Data analysis

Summary statistics are presented of all the variables. Categorical data are summarised using frequency counts or percentages, and distributions of variables are presented as two-way tables or bar charts. The mean was used as the measure of central tendency and standard deviation (s.d.) as the measure of spread. The prevalence of significant abnormal features on CT scan was calculated using the standard formula. Data were analysed by the research team using Microsoft Excel and SPSS statistical software (IBM Corp. Released 2020. IBM SPSS Statistics for Windows, Version 27.0. Armonk, NY: IBM Corp.).

### Ethical considerations

This study did not involve direct or indirect patient care and therefore posed minimal risk to patients. Identifiable data were removed as soon as the data collection for each patient was completed. A waiver of informed consent was approved, and the privacy and confidentiality of study subjects were ensured by utilising a password-protected computer and coding of the data points immediately after collection.

Institutional approval was obtained from Khayelitsha Hospital (Reference number: WC_202002_015) after the Health Research Ethics Committee of Stellenbosch University (Reference Number N19/10/131) approved the study.

## Results

A total of 3294 CT scans were documented by the radiology department at Khayelitsha Hospital over the 12-month study period. Fifty-two scans were excluded (duplicated reports *n* = 40, no report or no clinical information available *n* = 12) and 3242 CT scans were thus analysed.

The mean age of patients scanned was 45.6 years (s.d. = 17.6) and 51.4% (*n* = 1666) were males. Most CT scans involved a single body region (*n* = 2940, 90.7%), were contrasted (*n* = 2639, 81.4%) and were performed on an inpatient basis (*n* = 2608, 80.4%). There were 250 working days in 2019 with a mean of 12.9 scans performed per working day (3236/250; s.d. = 3.5); six scans were performed during weekend hours. The number of scans performed per weekday was equally distributed (Table 1).

**TABLE 1:** Overview of computed tomography utilisation across various parameters at Khayelitsha Hospital over a one-year period (*N* = 3242 unless specified otherwise).

Parameter	*n*	%	Mean	s.d.
**Age (years)**	-	-	45.6	17.6
**Gender**				
Female	1576	48.6	-	-
Male	1666	51.4	-	-
**Single versus multiple body regions**				
Single	2940	90.7	-	-
Multiple	302	9.3	-	-
**Contrast given**				
Contrasted	2639	81.4	-	-
Non-contrasted	603	18.6	-	-
**Inpatient versus outpatient**				
Inpatient	2608	80.4	-	-
Outpatient	634	19.6	-	-
**Day of week**				
Monday	636	19.6	-	-
Tuesday	656	20.2	-	-
Wednesday	681	21.0	-	-
Thursday	663	20.5	-	-
Friday	600	18.5	-	-
Saturday	3	0.1	-	-
Sunday	3	0.1	-	-
**Body area scanned (*N* = 3523)**				
Head and neck	1841	52.3	-	-
Chest	755	21.4	-	-
Abdomen and pelvis	673	19.1	-	-
Extremities	254	7.2	-	-
**Indication category for CT scan**				
Diagnostic: medical	2151	66.4	-	-
Diagnostic: malignancy	538	16.6	-	-
Pre-op planning	234	7.2	-	-
Diagnostic: trauma	160	4.9	-	-
Staging	130	4.0	-	-
Interval	29	0.9	-	-

s.d., standard deviation; CT, computed tomography.

The head and neck area were the most frequently scanned body region (*n* = 1841, 52.3%) and was substantially more than the chest (*n* = 755, 21.4%) and the abdomen and pelvis (*n* = 674, 19.1%) (Table 1). The indications for CT scan were mostly for the work up of general medical conditions totalling 2151 (66.4%) of all scan requests. This includes work up of cerebrovascular accidents, delirium, seizure disorders and cardio-respiratory diseases. A total of 538 (16.6%) scans were requested for suspected malignancy and 160 (4.9%) for the evaluation of traumatic injuries (Table 1).

Table 2 summarises the distribution of departmental scan requests per patient status. Most of the scans were requested by the Emergency Medicine department (*n* = 1382, 42.6%) and the Department of Internal Medicine (*n* = 695, 21,4%). Externally requested CT scans are those scans that were booked by the local clinics and 92.5% of externally requested scans were performed on an outpatient basis (Table 2). Emergency medicine and internal medicine requested the overwhelming majority of their CT scans as inpatients (95.9% and 93.1%, respectively). All CT scans requested by psychiatry were as inpatients because of the need to exclude a general medical condition as the cause of an acute psychosis.

**TABLE 2:** Computed tomography scans performed per department per patient status at Khayelitsha Hospital Hospital over a 1-year period. (*N* = 3242)

Department	Inpatient		Outpatient		Total	
	*n*	%	*n*	%	*N*	%
Emergency centre	1325	95.9	57	4.1	1382	42.6
External	18	7.5	222	92.5	240	7.4
Medicine	647	93.1	48	6.9	695	21.4
Obstetrics and gynaecology	12	75.0	4	25	16	0.5
Orthopaedics	105	46.7	120	53.3	225	6.9
Paediatrics	8	50.0	8	50.0	16	0.5
Psychiatry	124	100	0	0.0	124	3.8
Surgery	369	67.8	175	32.2	544	16.8

Most CT scans demonstrated some abnormality (*n* = 2710, 83.6%); however, only 2115 (65.2%) were deemed to have had a ‘positive yield’ (Table 3). Requests from the orthopaedic department had the highest positive diagnostic yield (96.4%) because most orthopaedic requests were for pre-operative planning. Surgery had an 80.9% positive yield, whereas Internal Medicine and Emergency Medicine had a positive yield rate of 67.5% and 60.2%, respectively (Table 3).

**TABLE 3:** Computed tomography scans per department by abnormality and yield performed at Khayelitsha hospital over a 1-year period.

Department	Abnormality				Yield				Total (*n*)
	Yes		No		Positive		Negative		
	*n*	%	*n*	%	*n*	%	*n*	%	
Emergency centre	1134	82.1	248	17.9	832	60.2	550	39.8	1382
External	152	63.3	88	36.7	116	48.3	124	51.7	240
Medicine	624	89.8	71	10.2	469	67.5	226	32.5	695
Obstetrics and gynaecology	13	81.2	3	18.8	11	68.8	5	31.2	16
Orthopaedics	217	96.4	8	3.6	217	96.4	8	3.6	225
Paediatrics	5	31.2	11	68.8	2	12.5	14	87.5	16
Psychiatry	62	50.0	62	50.0	28	22.6	96	77.4	124
Surgery	503	92.5	41	7.5	440	80.9	104	19.1	544

The CT diagnoses according to ICD-10 categories are presented in Table 4. To note is that 67% (71/106) of scans for a potential pulmonary embolism were positive. Aside from the normal and negative yield (non-diagnostic) scans, the greatest proportion of diagnoses were related to neoplasms (*n* = 467, 14.4%), circulatory (*n* = 451, 13.9%) and infectious (*n* = 419, 12.9%) (Table 4). The secondary diagnoses on CT scan are also listed in Table 4.

**TABLE 4:** Primary and secondary diagnosis on computed tomography scans performed at at Khayelitsha Hospital hospital over a 1-year period.

Primary diagnosis (ICD-10 code) (*N* = 3242)	*n*	%
I Certain infectious and parasitic diseases	419	12.9
II Neoplasms	467	14.4
III Diseases of the blood and blood-forming organs and certain disorders involving the immune mechanism	3	0.1
IV Endocrine, nutritional and metabolic diseases	6	0.2
V Mental and behavioural disorders	14	0.4
VI Diseases of the nervous system	165	5.1
VII Diseases of the eye and adnexa	3	0.1
VIII Diseases of the ear and mastoid process	3	0.1
IX Diseases of the circulatory system	451	13.9
X Diseases of the respiratory system	151	4.7
XI Diseases of the digestive system	141	4.4
XII Diseases of the skin and subcutaneous tissue	5	0.2
XIII Diseases of the musculoskeletal system and connective tissue	30	0.9
XIV Diseases of the genitourinary system	34	1.1
XVII Congenital malformations, deformations and chromosomal abnormalities	4	0.1
XIX Injury, poisoning and certain other consequences of external causes	353	10.9
Non-diagnostic	458	14.1
Normal	535	16.5
**Secondary diagnosis (ICD-10 code) (*N* = 42)**		
I Certain infectious and parasitic diseases	8	19.1
II Neoplasms	3	7.2
VI Diseases of the nervous system	2	4.8
IX Diseases of the circulatory system	8	19.1
X Diseases of the respiratory system	3	7.1
XI Diseases of the digestive system	4	9.5
XII Diseases of the skin and subcutaneous tissue	1	2.4
XIII Diseases of the musculoskeletal system and connective tissue	1	2.4
XIX Injury, poisoning and certain other consequences of external cause	5	11.9
Non-diagnostic	3	7.1
Normal	4	9.5

ICD-10, International Statistical Classification of Diseases and Related Health Problems, 10th Revision.

There was congruency between the clinical (pre-CT) and final CT diagnoses in 1610 (49.7%) cases, 1629 (50.2%) were not congruent and agreement was not applicable in three (0.1%) cases.

## Discussion

This study, exploring the utilisation of CT in South Africa, was unique from its point of view at the district level. However, the specific objectives of describing the CT types, the clinical indications, assessing the overall diagnostic yield and clinical impact improves our understanding of appropriate CT utilisation in general, regardless of the level of care. Furthermore, it may also offer valuable insights for enhancing efficiency and overall patient care across diverse healthcare settings.

The Canadian Institute for Health Information (CIHI) reported a national average of three CT scans per hour per scanner in 2005.^15^ This was almost double the average of 1.6 scans per hour in this study; however, the differences in infrastructure are stark. The reason for the lower number of scans per hour could be multifactorial, especially in our setting where the CT suite is a relatively new service. Infrastructure challenges, such as an inadequate electricity supply and unstable internet connectivity coupled with limited maintenance and servicing options lead to intermittent breakdowns and downtime of CT services. In addition, a lack of trained personnel including dedicated porters, nurses and radiographers are just some factors that can hinder the effective operation of CT scanners. An outpatient facility in the United States embarked on a process to maximise CT productivity.^16^ The study indicated that a single technologist (radiographer) needed to complete up to 34 tasks per patient, resulting in only scanning 2.2 patients per hour.^16^ The number of CT scans performed per hour increased substantially to 5.2 when there were two technologists per patient. It increased further to 7.5 when there were three technologists per patient.^16^

Another notable difference was in the indications for CT, which indicated that only 4.9% of indications were related to trauma, whereas in another South African study, 76% of CT scans were trauma related.^6^ Although the latter study was performed in a tertiary hospital, the main reason for the low percentage of trauma-related CT scans at Khayelitsha Hospital was because of the policy that was in place. At the time of the study, all trauma patients presenting to Khayelitsha Hospital with an indication for a CT scan were transferred to the local tertiary hospital.

The diagnostic yield of 65%, as determined in our study, aligns closely with findings from both local and international research. A Johannesburg-based study conducted in an emergency centre of a tertiary hospital previously reported a comparable positive yield of 61%,^6^ indicating consistency in diagnostic outcomes across different healthcare settings. Similarly, a Malaysian study focusing on CT scans of the head exhibited an overall positive yield of 65.8%, with a higher positive yield (71%) for non-trauma cases.^5^

Furthermore, delving into the yield of specific CT scans, recent research at the University of Iowa hospitals examined the positive yield rate of CT pulmonary angiograms in detecting pulmonary embolism. The reported yield of 10.9% was considered comparable to existing literature and deemed an acceptable positivity rate.^17^ In contrast, our study revealed a substantially higher positive yield rate of 67%, with 71 scans indicating pulmonary embolism out of a total of 106 requests over the 1-year period. This discrepancy may reflect the scarcity of resources and potentially indicate an underutilisation of CT scans compared to the international norm.

Half of the performed CT scans were congruent between the initial clinical impression and the CT diagnosis. Pescatori et al. found a similar congruency ratio in an Italian study. Their study evaluated the clinical effect of CT in non-traumatic chest and abdominal scans, and the clinical hypotheses were confirmed in 45% of cases while there was a major variation in diagnoses in 37% of cases.^4^ However, a normal CT scan does not imply the test was unnecessary, and similarly, non-congruency between pre- and post-CT diagnoses is not necessarily an indication of an unnecessarily performed test. Computed tomography scans are often ordered particularly to exclude an alternate or underlying diagnosis. An example is in the workup of patients with new onset seizures; a normal CT scan will have a negative yield and be non-congruent, but a very necessary diagnostic test to exclude potentially treatable causes. Similarly, young patients with mental health illnesses undergoing CT to exclude a delirium or patients newly diagnosed with cancer getting a metastatic workup, etc. It is in these cases, where the basis of the CT is to exclude a diagnosis, that the congruency may become skewed. The requesting information lists the alternate diagnosis as the one under investigation and thus in these cases, a normal scan will be non-congruent even though there may have been a low level of true clinical suspicion.

This study’s findings highlight several areas for potential improvement in patient care at both the facility and broader governmental policy levels. As mentioned, the comparatively low number of scans per day and per hour could imply that the efficiency of the system could be improved. Introducing additional personnel or implementing standardised procedures to optimise the process may aid in increasing efficiency.

The majority of scans (over 90%) requested by internal medicine and emergency medicine were for inpatients. This prompts the question of whether all these scans were necessary for inpatient care, or if there’s a need for a more efficient outpatient system for bookings and follow-ups. Keeping well patients in hospital just to get a CT scan should be a contributing factor to the overburdening of inpatient systems and might aggravate bed shortages.

Finally, hospital policy at the time resulted in the unexpectedly low number of trauma scans being performed in a community with a high trauma burden.^12^ This is a contentious issue that needs further evaluation. Performing more trauma-related CT scans at the district level could alleviate the strain on emergency medical services to transport patients only for a diagnostic study. Similarly, the receiving facility can also be negatively impacted by the greater caseload of patients requiring further investigation. On the other hand, upscaling the CT service to provide a 24/7 service will be costly to the district hospital and is sure to increase the burden on an already over-burdened system. Certain specialist services, particularly neurosurgery, are also not available at the district level and a delay in definitive treatment might be detrimental to patients. A birds-eye evaluation is needed to create an optimal system that ensures cost-effective care without overburdening one facility at the expense of another.

### Limitations

This study was limited to a single facility and the results might not be generalisable. This was also a retrospective study, and there was no reliable method to assess the accuracy of requesting information. Furthermore, clinical notes were not easily accessible and often missing from the hospital electronic record system. Therefore, the clinical information was obtained solely from the CT request form.

Diagnostic codes (ICD-10) were used as a surrogate measure, and we did not attempt to quantify any bias that may have arisen from misclassifications surrounding the accuracy of the diagnosis or the correlation between the diagnostic code and the documented diagnosis.

The proportion of scans requested from the emergency centre might be overestimated. In early 2019, any hospital physician could request a CT under the emergency centre’s profile, which may have been used by other departments when requesting a CT. However, the potential effect was minimised by scrutinising the clinical history and patient location in the hospital. This often revealed the correct requesting department.

## Conclusion

The utilisation of CT scans at district-level healthcare facilities yields comparable positive rates to those observed in tertiary centres. This suggests that the service is appropriately utilised and potentially alleviating the burden on higher-level referral facilities. Overall, regionalisation of CT utilisation holds great potential to enhance healthcare quality and accessibility in district-level health facilities, facilitating a more equitable distribution of advanced diagnostic services, ensuring that patients in underserved areas have access to crucial imaging technology and ultimately promoting better patient outcomes. However, the relatively low number of CT scans conducted per hour indicates a complex issue influenced by multiple factors. Infrastructure challenges (e.g. inadequate electricity supply, unstable internet connectivity), limited maintenance and servicing options and a lack of trained personnel are just some barriers to consider.

Further research and dialogue are warranted to refine policies, enhance efficiency and improve patient care in the context of diagnostic imaging at the district level in South Africa and similar settings.

## References

[1] Flohr T. CT systems. Curr Radiol Rep. 2013;1(1):52–63. 10.1007/s40134-012-0005-5

[2] Bellolio MF, Heien HC, Sangaralingham LR, et al. Increased computed tomography utilization in the Emergency Department and Its Association with Hospital Admission. West J Emerg Med. 2017;18(5):835–845. 10.5811/westjem.2017.5.34152 28874935 PMC5576619

[3] Becker J, Jenkins LS, De Swardt M, Sayed R, Viljoen M. Appropriateness of computed tomography and magnetic resonance imaging scans in the Eden and Central Karoo districts of the Western Cape Province, South Africa. S Afr Med J. 2014;104(11):762. 10.7196/SAMJ.8158 25909118

[4] Pescatori LC, Brambati M, Messina C, et al. Clinical impact of computed tomography in the emergency department in nontraumatic chest and abdominal conditions. Emerg Radiol. 2018;25:393–398. 10.1007/s10140-018-1592-0 29536277

[5] Ismail MS, Shahridan MF, Hong SB, Azhar AA, Bener A. Emergency head CT scan ordering and yield: A Malaysian experience. J Emerg Med Trauma Acute Care. 2008;8(3):151–155.

[6] Swartzberg K, Goldstein LN. High positive computed tomography yields in the emergency department might not be a positive finding. S Afr Med J. 2018;108(3):230–234. 10.7196/SAMJ.2018.v108i3.12635 30004368

[7] Kabongo JM, Nel S, Pitcher RD. An analysis of licensed South African diagnostic imaging equipment. Pan Afr Med J. 2015;22:57. 10.11604/pamj.2015.22.57.7016 26834910 PMC4725661

[8] Makanjee CR, Bergh AM, Hoffmann WA. A model for understanding diagnostic imaging referrals and complex interaction processes within the bigger picture of a healthcare system. Radiography. 2014;20(2):153–157. 10.1016/j.radi.2014.01.005

[9] Bergeron C, Fleet R, Tounkara FK, Lavallée-Bourget I, Turgeon-Pelchat C. Lack of CT scanner in a rural emergency department increases inter-facility transfers: A pilot study. BMC Res Notes. 2017;10(1). 10.1186/s13104-017-3071-1 PMC5745590 29282113

[10] Sokanyile A. ‘Khayelitsha Hospital’s CT scan suite a first’. Weekend Argus [serial online], 2018 Aug; p. 6 [cited 2023 Dec 10]. Available from: https://www.pressreader.com/south-africa/weekend-argus-saturday-edition/20180804/281642485986942

[11] Strategic Development Information and GIS Department (SDI&GIS). City of Cape Town – 2011 Census – Khayelitsha Health District. August, 2013; pp. 1–7 [cited 2023 Nov 20]. Available from: https://resource.capetown.gov.za/documentcentre/Documents/Maps%20and%20statistics/Khayelitsha%20Health%20District.pdf

[12] Hunter LD, Lahri S, Van Hoving DJ. Case mix of patients managed in the resuscitation area of a district-level public hospital in Cape Town. Afr J Emerg Med. 2017;7(1):19–23. 10.1016/j.afjem.2017.01.001 30485864 PMC6234138

[13] Swarts L, Lahri S, Van Hoving DJ. The burden of HIV and tuberculosis on the resuscitation area of an urban district-level hospital in Cape Town. Afr J Emerg Med. 2021;11(1):165–170. 10.1016/j.afjem.2020.09.016 33680739 PMC7910156

[14] Western Cape Government. Eco-friendly Khayelitsha Hospital [homepage on the Internet]. [cited 2019 Aug 25]. Available from: http://www.westerncape.gov.za/general-publication/eco-friendly-khayelitsha-hospital

[15] Ariste R, Fortin G. Could MRI and CT scanners be operated more intensively in Canada? Healthc Policy. 2007;3(1):e113–e120. 10.12927/hcpol.2007.19141 19305746 PMC2645123

[16] Boland GWL, Houghton MP, Marchione DG, Mccormick W. Maximizing outpatient computed tomography productivity using multiple technologists. J Am Coll Radiol. 2008;5(2):119–125. 10.1016/j.jacr.2007.07.009 18242528

[17] Aggarwal T, Eskandari A, Priya S, et al. Pulmonary embolism rule out: Positivity and factors affecting the yield of CT angiography. Postgrad Med J. 2020;96(1140):594–599. 10.1136/postgradmedj-2019-137031 31907225

